# Complete Genome Sequence of Salmonella enterica Serovar Typhimurium Siphophage Shivani

**DOI:** 10.1128/genomeA.01443-14

**Published:** 2015-02-26

**Authors:** Denish Piya, Yicheng Xie, Adriana C. Hernandez Morales, Gabriel F. Kuty Everett

**Affiliations:** Center for Phage Technology, Texas A&M University, College Station, Texas, USA

## Abstract

Here, we describe the complete genome sequence of siphophage Shivani, a T5-like constituent phage in the therapeutic phage cocktail IntestiPhage developed for bacterial gastroenteritis. Shivani was isolated against a foodborne pathogen, Salmonella enterica, which is one of the leading causes of gastroenteritis.

## GENOME ANNOUNCEMENT

Salmonella enterica serovar Typhimurium infects humans and other mammalian species and is a leading cause of gastroenteritis, primarily from the consumption of contaminated food (1). IntestiPhage (developed by the George Eliava Institute of Bacteriophages, Microbiology, and Virology, Tbilisi, Georgia) is a therapeutic product against several gastroenteritis-causing bacteria. IntestiPhage consists of 23 different phages targeting various enteric pathogens (2). Here, we present the complete genome sequence of one of the component phages of IntestiPhage, a T5-like siphophage, Shivani.

Bacteriophage Shivani was isolated from IntestiPhage (lot no. M2-401). Phage DNA was sequenced in an Illumina MiSeq 250-bp paired-end run with a 550-bp insert library at the Genomic Sequencing and Analysis Facility at the University of Texas (Austin, TX). Quality-controlled trimmed reads were assembled to a single contig at 75-fold coverage using Velvet version 1.2.10. The contig was confirmed to be complete by PCR using primers that face the upstream and downstream ends of the phage DNA. The products from the PCR amplification of the junctions of concatemeric molecules were sequenced by Sanger sequencing (Eton Bioscience, San Diego, CA). Genes were predicted using GeneMarkS (3) and corrected using software tools available on the Center for Phage Technology (CPT) Galaxy instance (https://cpt.tamu.edu/galaxy-public/). Morphology was determined using transmission electron microscopy performed at the Texas A&M University Microscopy and Imaging Center.

Shivani is a siphophage with a unit genome of 109,193 bp containing 155 putative genes (1). It shares 73.4% sequence identity with Escherichia coli phage T5 (accession no. NC_005859). Shivani has a G+C content of 38.4%, which is significantly lower than the 53.33% G+C content of the host. This is comparable to the 39.2% G+C content of T5. An 11,123-bp long terminal repeat was identified using the PAUSE method (https://cpt.tamu.edu/computer-resources/pause/) on the raw sequencing data. Shivani encodes 18 putative HNH endonucleases compared to the 8 putative HNH endonucleases encoded by T5.

Overall, Shivani is syntenic with T5, with most of the differences between the phages occurring in hypothetical conserved or novel genes of unknown function. It contains core T5-like genes encoding proteins corresponding to host shutdown, lysis, DNA replication/recombination, regulation (with the exception of a Sir2-like regulator protein), biosynthesis, and morphology functions. Unlike T5, the alpha subunit of the ribonucleoside-diphosphate reductase of Shivani is interrupted by an intein. Based on BLASTp homology and sequence analysis, the boundaries of the intein were determined to be Cys292 and Asn595 (4, 5). To accomplish lysis, Shivani uses a T5-like lysis cassette including a holin, soluble endolysin (peptidase), and inner and outer membrane spanins (6). The spanin layout of Shivani, however, is different from that of T5, in that Shivani has a hypothetical conserved gene of unknown function separating the two lysis proteins.

Interestingly, Shivani is missing the T5-like lytic conversion lipoprotein precursor (*llp*) that blocks the FhuA receptor of the host to prevent readsorption of the progeny phage to cell debris after lysis (7). Presumably, this protein would be a great benefit to a bacteriophage used for therapy applications to prevent the inactivation of the progeny phage.

### Nucleotide sequence accession number.

The genome sequence of phage Shivani was contributed to GenBank as accession no. KP143763.
